# Beyond the surface: diagnosing the origins of institutional barriers in the governance of floating offshore wind

**DOI:** 10.1007/s40152-026-00499-4

**Published:** 2026-06-30

**Authors:** Lindsey West, Christina Kelly, Wesley Flannery

**Affiliations:** School of Natural and Built Environment, Queen’s University Belfast, Belfast, BT9 5AG UK

**Keywords:** Marine governance, Institutional attributes, Institutional barriers, Fragmentation, Floating wind, Celtic Sea

## Abstract

The expansion of floating offshore wind in the UK’s Celtic Sea presents a major opportunity to accelerate the low-carbon transition while supporting regional economic development. However, its implementation is unfolding within a fragmented governance landscape, which constrains the development of new governance approaches that can deliver on multiple policy objectives, including net-zero, nature recovery, and economic growth. Fragmentation is a well-documented feature of marine governance systems and is commonly associated with institutional barriers such as path dependency, policy layering, and institutional inertia. However, few studies have examined the underlying causes of these barriers, meaning that resulting recommendations often fail to tackle foundational institutional design issues. Drawing on institutional theory and using a diagnostic analytical approach, this study identifies multiple institutional barriers that hinder the development of floating wind in the Celtic Sea. The barriers are traced to underlying institutional attributes, including those that regulate actor roles and responsibilities, structure social networks, and distribute power and authority. The paper concludes that the governance challenges observed in the UK’s Celtic Sea arise from systemic and synergistic institutional barriers rather than as a result of isolated policy failures. Therefore, overcoming institutional barriers demands a reconfiguration of marine governance arrangements to enhance reflexivity, adaptability, and collaborative learning across governance scales and policy domains. By linking the emergence of institutional barriers to underlying institutional attributes, the paper contributes to the literature on institutional dynamics in marine governance and highlights the need for new marine governance arrangements that can deliver a coordinated and holistic approach to marine policy and support a just and equitable transition to a low-carbon society.

**Supplementary Information:**

The online version contains supplementary material available at https://doi.org/10.1007/s40152-026-00499-4.

## Introduction

The transition to a low-carbon society requires innovative marine governance arrangements that can support offshore renewable energy (ORE) development while balancing multiple and often intersecting marine policy objectives. Changing ocean conditions, induced by climate change, also necessitate the adoption of future-oriented and anticipatory governance approaches (Frazão Santos et al. 2024). However, current marine governance arrangements are ill-suited to meet these new demands due to high levels of fragmentation (Grip 2017), deeply entrenched power asymmetries (Keijzer et al. 2024), and the politicised nature of ocean space (Bennett 2019). These issues present persistent challenges for policymakers and reflect the complex institutional dynamics that underpin marine governance (Kelly et al. 2018). Complexity is embedded in the low-carbon transition due to the cross-cutting nature of ORE development. The involvement of a wide range of actors, including policymakers, regulators, industry representatives, and diverse marine users, means that cooperation and coordination across scales, sectors, and policy domains are essential, while decision-makers must also navigate complex and often problematic power relations that characterise energy systems (Avelino et al. 2023). Thus, ORE expansion depends not only on technological capability but on the capacity of institutions to innovate amid a complex and rapidly changing environment, and at the same time, function effectively to maintain legitimacy (Radtke 2025). In view of this tension, and as ocean space becomes increasingly congested, there is a pressing need to understand how the attributes of institutions enable and constrain governance innovation in response to emerging challenges, including the low-carbon transition.

Institutional attributes are the formal and informal features, characteristics, or properties of institutions that shape how they function and how they influence governance arenas, including their structures, rules, decision-making processes, and resources (Young 2002; Ostrom 2005). Well-designed attributes can enhance governance performance, exemplified by recent developments in Northern Ireland’s fishing sector, following the establishment of the ‘Co-Fish’ network in 2022. Institutional innovations have transformed how governance actors collaborate, share knowledge, and contribute to policymaking on fisheries management, leading to enhanced social learning and a notable shift in power relations amongst governance actors (McAteer and Flannery 2025). Similarly, in multi-level governance systems, well-designed institutional attributes can foster linkages amongst actors across scales (Cash et al. 2006), resulting in increased trust and cooperation, as well as opportunities for learning and experimentation (Carlisle and Gruby 2019). However, governance arrangements are typically embedded in long-standing institutional choices that are designed to promote stability and are therefore highly resistant to change (Allen et al. 2023). In these circumstances, institutional attributes can give rise to institutional barriers, understood as rules, norms, or structural features within an institution that impede progress towards desired governance outcomes (Ostrom 2005; Young 2010). For example, Turner et al. (2020) found that institutional attributes designed to support new co-management approaches in the Caribbean were poorly suited to fostering vertical connections between centralised governance structures and island communities, creating institutional barriers that hindered information sharing and limited local participation in coral reef governance. Taken together, these two examples highlight the dual function of institutional attributes, in that they can both enable and constrain governance change and innovation (Adjei et al. 2026).

An expanding body of scholarship has drawn attention to persistent institutional barriers in marine governance. Several studies have shown how institutional barriers limit the scope for implementing new policy approaches, leading to a range of negative impacts, including contested access to marine resources (Ansong et al. 2022); tensions between actors at different tiers of governance (Greenhill et al. 2020); and weakened public trust in authorities and the wider planning system (Kelly et al. 2019). Other studies have shown that institutional barriers obstruct knowledge-sharing pathways (e.g. Weiss et al. 2012; Cvitanovic et al. 2015; Morf et al. 2023), thereby constraining the social learning and reflexivity needed to foster innovative governance approaches (Schutter et al. 2025). Institutional barriers also limit meaningful public engagement in marine governance by sustaining top-down processes (Flannery et al. 2018; Tafon et al. 2023; Wilke 2023). These kinds of power asymmetries act as institutional barriers to fair, just, and equitable marine governance, particularly where dominant economic actors are prioritised over marginalised groups such as small-scale fishers and Indigenous communities (Jentoft and Chuenpagdee 2009; Bennett et al. 2019; Parsons et al. 2021).

Although these studies reveal the pervasive impacts of institutional barriers, there remains a paucity of research on the origins of institutional barriers in marine governance and their relationship with particular institutional attributes. The paper addresses this empirical gap by identifying institutional barriers that hinder governance change and innovation in the UK’s Celtic Sea, where a new floating offshore wind (FLOW) industry is being established. By adopting a novel diagnostic approach, we trace patterns of persistent problems in marine governance to key institutional attributes. Drawing on institutional theory, the paper begins with a review of common institutional barriers in marine governance, showing how they arise from maladapted institutional attributes and operate synergistically to impede change. This is followed by a description of the methodological approach adopted in this study. The research findings are presented as three case-based examples of governance challenges that are linked to a range of institutional attributes, including those that regulate actor roles and responsibilities, allocate power and authority amongst actors, and shape how knowledge and information are constructed, accepted, and communicated. The paper concludes with a discussion on the potential for governance change and innovation in the UK’s Celtic Sea in light of the study’s findings, together with a reflection on the value of a diagnostic approach for understanding institutional barriers in marine governance.

### Institutional attributes and barriers in marine governance

Institutions are understood as *“a relatively stable collection of practices and rules defining appropriate behaviour for specific groups of actors in specific situations”* (March and Olsen 1998, p.948). By defining the behavioural norms that shape behaviour, institutions make cooperation and collective action possible (Klijn and Koppenjan 2006). Despite their relative stability, institutions change over time as networks of actors work to construct, maintain, or disrupt institutional structures through their agency and interactions (Beunen and Patterson 2019). At the same time, rigid institutional structures and the ‘rules-in-use’ may constrain the agency of actors to enact change (Werbeloff et al. 2016). Thus, governance arrangements are continuously formed, negotiated and (re)produced through a process of structuration, reflecting the dynamic interplay between agency and structure (van Noort et al. 2025). Structuration creates the conditions under which institutional attributes emerge, including the rules that define who is eligible to participate in governance, which actions are permitted, and how collective decisions are made (Ostrom 2005). Stability-enhancing attributes, such as formal rules, organisational hierarchies, and established coordination structures, create predictability, reduce uncertainty, and enable institutions to stabilise and endure over time. In contrast, informal norms and practices, discretionary authority, and feedback and learning mechanisms can introduce flexibility and provide opportunities for institutional adaptation. Thus, governance change and innovation emerge from ongoing processes of structuration and stabilisation, implying that governance arrangements are always in flux (van Tatenhove 2022).

The design of institutional attributes shapes the capacity of governance arrangements to change, innovate, and achieve successful outcomes. With a focus on environmental governance for sustainability, Schoon and Cox (2018) highlight the importance of attributes that support collaboration amongst governance actors, enable working across scales (vertically and horizontally), and allow institutional adaptation and evolution. In the face of new and emerging marine governance challenges, institutional attributes that support social learning and reflexivity are crucial for achieving desired governance outcomes (van Leeuwen et al. 2025). Of central importance in all governance contexts are institutional attributes that distribute power and authority appropriately, as they ensure that decision-making is both legitimate and responsive to a particular governance context (Fudge et al. 2021). Each of these attributes is included in a taxonomy of 10 institutional attributes (see Table 1) that were identified as important for shaping adaptation to climate change in Europe (Oberlack 2017). The taxonomy conceptually organises institutional attributes across three key dimensions of agency, social interactions, and inherent attributes of institutions, thereby capturing how processes of structuration and stabilisation shape adaptation outcomes. Nielsen et al. (2023) extended the taxonomy following their scoping review of institutional attributes and barriers in a European maritime context and further highlighted the dual function of institutional attributes in enabling and constraining governance change and innovation.

When political priorities shift or new governance challenges emerge, well-designed institutional attributes can become maladapted and give rise to institutional barriers. Maladaptations commonly occur when new rules and approaches are incorporated into existing policy frameworks or systems of power (Kelly et al. 2018), leading to mismatches between institutional design and institutional objectives (Fullbrook and Vince 2026). Such mismatches limit the capacity of governance arrangements to respond at the appropriate scale and speed, resulting in outdated policies and regulatory frameworks that lack legitimacy. Policy layering, path dependency, and bounded rationality are examples of persistent institutional barriers that inhibit transformative marine governance (Kelly et al. 2019). These barriers align with stability-oriented institutional attributes, such as rigid formal rules, entrenched organisational hierarchies, and routinised coordination mechanisms. Together, these attributes privilege established actors, reinforce dominant norms, and marginalise alternative knowledge and perspectives, making it difficult to challenge or transform existing governance arrangements. Policy-layering occurs when new institutions or mandates are ‘bolted-on’ to existing policy regimes without removing others (van der Heijden 2011). Governance actors who desire change may be constrained by rigid institutional structures or they may lack capacity to alter the original rules, and instead, resort to amending or modifying existing elements of the rules-in-use, with small changes accumulating over time (Mahoney and Thelen 2010). While layering is often understood as a driver of gradual policy change, it can also reproduce a specific configuration of institutions, thereby preserving the status quo and ‘locking-in’ existing institutional arrangements (Capano 2019). In an analysis of the EU’s Integrated Maritime Policy, Paridaens and Notteboom (2021) concluded that the policy had been developed through a gradual process of (re)combining and layering a diverse range of sector-based strategic documents and tools, leaving deep-rooted policy trajectories largely intact. Policy layering can be an intentional policy choice in pursuit of stability (Capano 2019), but it is often a response to path-dependency, in which current decisions are shaped by old narratives, historical decisions, or outdated knowledge, thereby restricting the range of policy options that can be considered (Barnett et al. 2015). In this situation, existing policies become difficult to dismantle, so new policies are layered on top, yet new policy options tend to mirror established practices or past decisions (Kirk et al. 2007). Layering can reinforce path-dependency as each new layer creates additional complexity and the behaviour of governance actors becomes structured by established norms, rules, and routines, eventually becoming locked-in due to the economic and political costs of deviating from the chosen path (Peters et al. 2005). Path-dependency leads to slow and incremental change and risks further entrenching existing power dynamics and vested interests (Evans et al. 2023). In a marine governance context, path dependency has had wide-ranging impacts, including hindering the establishment of socially just forms of marine governance (Talib et al. 2022); impeding adaptation to climate change in coastal cities (Nunn et al. 2021); and contributing to fishery collapse (Bertheussen 2022).

Path dependency is also linked to bounded rationality, which takes account of the cognitive limitations of decision-makers in attempting to achieve their policy goals (Jones 1999). These limitations arise because governance actors rarely have access to the full information needed to implement a new policy approach (Kirk et al. 2007). Therefore, they make decisions based on incomplete information and adopt an incremental approach to gathering new information, often due to resource constraints. Moreover, governance actors are likely to have incurred considerable transaction costs in accumulating their knowledge and experiences, and hence, have vested interests in them (Kyriazis and Metaxas 2010). Governance fragmentation exacerbates bounded rationality because data and information that can be used to guide social learning and facilitate policy innovations are often dispersed across multiple organisations (Murray-O’Connor and Cooper 2024), while power asymmetries determine who has access to information (Mol 2006). Clarke and Flannery (2020) noted the existence of bounded rationality in the English marine planning system, whereby data gaps were acknowledged, but due to time and resource constraints, only superficial attempts were made to address them, which created an illusion of change and innovation against a background of path-dependency. The impacts of bounded rationality and path-dependency are also visible in the offshore wind consenting process, where industry regulators rarely adopt new modelling techniques to understand bird collision risk, despite their availability, and instead, continue to rely on outdated but familiar models, resulting in lengthy consenting processes or even the refusal of planning consent (Cook et al. 2025).

Institutional barriers can also work synergistically, further preserving stability by limiting opportunities for rule negotiation or reinterpretation, while reinforcing shared understandings of how the system works and where power lies within it. This synergistic behaviour may explain why efforts to improve the performance of marine governance arrangements have not always been successful (e.g. Kelly et al. 2019; Fullbrook et al. 2026). Furthermore, institutional barriers are rarely interpreted and experienced uniformly (Eisenack et al. 2014). For example, constraints on coordination may be perceived as advantageous by those with a vested interest in maintaining the status quo or protecting existing power relations. In a study of barriers to climate change adaptation in the Netherlands, Biesbroek et al. (2011) noted that perceptions of the severity of institutional barriers reflected power relations between national and local governance actors, with actors at higher tiers of governance who controlled the budgets for climate adaptation, regarding the barriers as less severe than local governance actors. Similarly, institutional barriers that contribute to weak climate policy or slow down policy implementation are advantageous for (multi)national oil and gas companies (Herzog-Hawelka and Gupta 2023). Thus, institutional barriers are relative to the governance context and scale, as well as the actors experiencing them. Tracing institutional barriers to specific institutional attributes can therefore assist with ‘diagnosing’ the causes of persistent governance challenges and uncover strategic leverage points for driving governance change and innovation.

### Study site and methodology

**Fig. 1:**
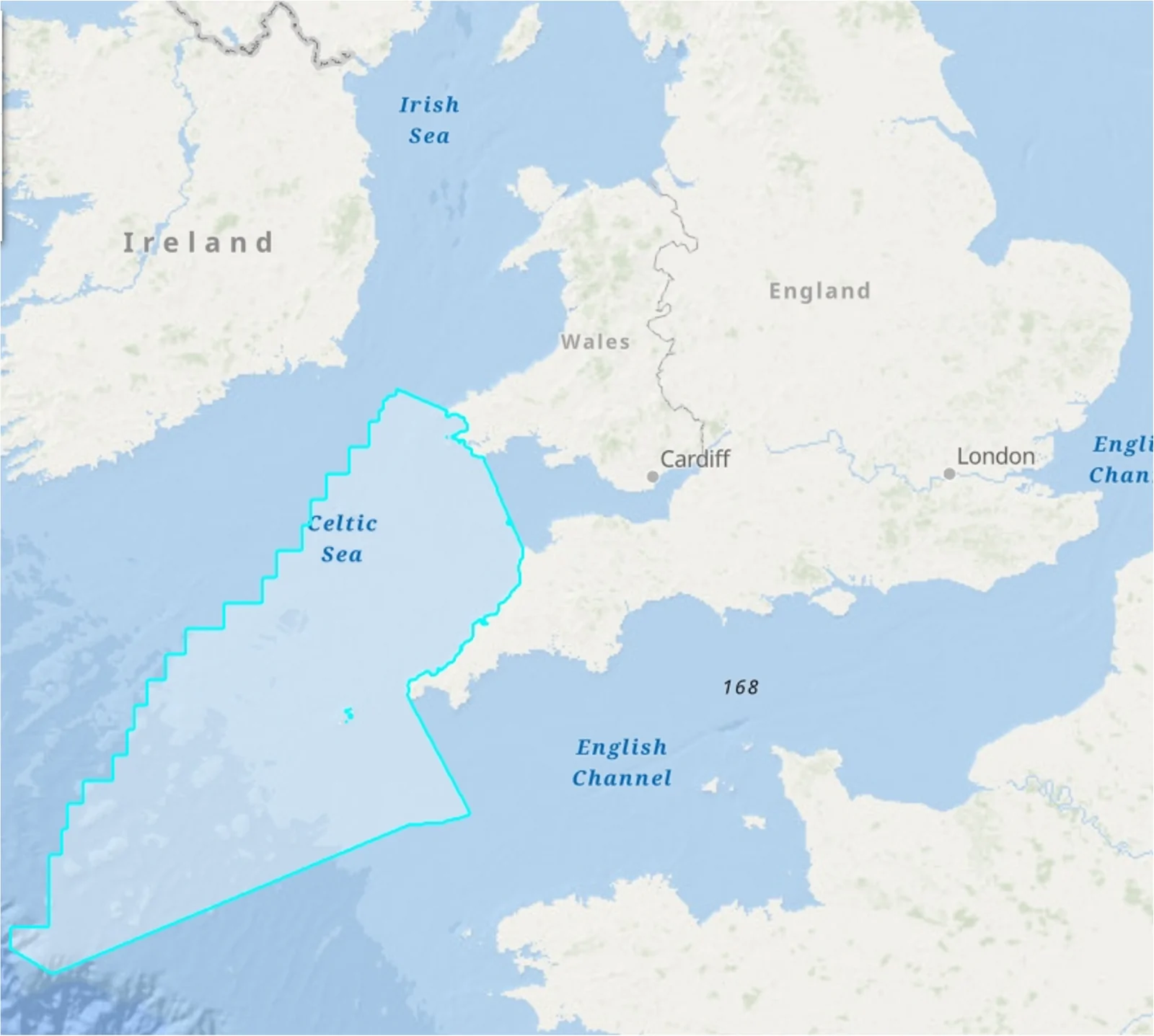
The UK’s Celtic Sea area. (Base layer: Oceans, source ESRI; Celtic Sea area, source the crown estate, Celtic Sea area of interest)

The UK’s Celtic Sea area (Fig. 1) was selected as a case study site due to the current development of 4.5GW of FLOW under The Crown Estate’s Celtic Sea Floating Offshore Wind Leasing Round 5 (The Crown Estate 2023). The establishment of a new FLOW industry in the Celtic Sea is intended to make a significant contribution to delivering the UK’s offshore wind ambitions of 43–50 GW by 2030 (DESNZ 2024) and broader 2050 net-zero targets. However, the Celtic Sea is subject to diverse sectoral demands (Welsh Government 2019; MMO 2021) and therefore presents a challenging context for the governance of a new FLOW industry. It is a marine biodiversity hotspot, supporting a network of marine protected areas as well as commercially important fish stocks, and it hosts some of the UK’s busiest ports and shipping lanes. Hence, FLOW development intersects with multiple policy domains, including climate, energy, fisheries, environment, trade, and defence. The case study context is further complicated by unique seabed ownership arrangements. The Crown Estate is the owner and manager of the seabed in England and Wales and is therefore an influential actor in FLOW development. The specific siting of offshore wind farms is determined by The Crown Estate through its seabed leasing process.

The study adopts a diagnostic approach by drawing on a typology of institutional attributes developed by Oberlack (2017) and extended by Nielsen et al. (2023). The 11 institutional attributes relevant to adaptation processes (Table 1) relate to the rules and procedures that shape the decision-making of individual governance actors (i.e. individual actor agency); the procedures and networks that link individual actors (i.e. social interactions between actors); and the inherent attributes of the institutions themselves. Thus, the interplay of agency and structure is embedded within the typology.

**Table 1:** Typology of institutional attributes in climate adaptation (from Oberlack (2017) and Nielsen et al. (2023)

Institutional attribute	Dimension
Actor eligibility	Individual agency of governance actors
Roles and responsibilities	
Control	
Accountability mechanisms	Social interactions between governance actors
Social connectivity	
Conflict mechanisms	
Social learning/Development and use of knowledge	
Scale of institutions	Inherent attributes of institutions
Adaptiveness of institutions	
Formality of institutions	
Institutionalised incentives	

Empirical data on institutional barriers were collected using a mixed-method approach, which combined document analysis, semi-structured interviews, and story-mapping. The document analysis involved a review of 57 policies, plans, technical reports, policy briefs, meeting presentations and minutes, parliamentary transcripts, select committee evidence (written and oral), position papers, and industry blogs (see Supplementary Information for full list). Documents were selected for analysis based on their relevance to marine governance and to FLOW development in the UK between 2021 and 2025. This timeframe was selected to take account of the publication of the UK’s first FLOW targets in the Net-Zero Strategy (BEIS 2021) and to ensure inclusion of new policies, plans, and reports emerging after the election of a new UK Government in July 2024. Inclusion criteria required that documents directly addressed marine governance and/or FLOW development, including but not limited to offshore wind planning and consenting, marine spatial planning, and stakeholder engagement. Materials that were purely technical or unrelated to marine governance were excluded.

A total of 52 FLOW stakeholders, identified using purposive sampling, were invited to participate in an online interview. Of those, 24 responded to the invite, and online interviews were conducted between May and September 2024 (Table 2). The interviews were guided by open-ended questions focusing on the factors that constrain the development of FLOW in the Celtic Sea. Interviews were recorded and transcribed, and the transcripts were analysed using thematic analysis (Boyatzis 1998) in NVivo software (version 1.3).

The two sets of data were analysed and interpreted together and treated equally to provide both breadth and depth. The document analysis was intended to identify a broad range of institutional barriers, while the interviews aimed to provide detailed insights into interviewees’ perspectives and experiences. The analysis was informed by an abductive approach (Timmermans and Tavory 2012). Deductive coding of the documents and interview transcripts was based on the typology of institutional attributes identified by Oberlack (2017) and Nielsen et al. (2023). References to additional institutional attributes were identified and coded inductively. Coding criteria focused on identifying references to regulatory constraints, coordination challenges, power asymmetries, and gaps between policy goals and practice as a way of making visible the specific rules, structures, and relations that hinder adaptation, collaboration, or policy implementation. This approach ensured that the analysis systematically captured how institutional barriers were represented and could be traced back to underlying institutional attributes set out in the Oberlack (2017) and Nielsen et al. (2023) typology.

In November 2024, a preliminary analysis of the data was shared with the same 52 FLOW stakeholders that were originally invited to participate in an interview. The analysis was presented in ArcGIS StoryMaps, a novel online communication tool that combines interactive maps and multimedia content to tell a particular story (Kerski 2019). A survey consisting of 10 statements (see Supplementary Information), each with a five-point Likert Scale response, was incorporated into the StoryMap. The purpose of the survey was to validate the preliminary analysis and gather further insights into perceptions of current governance innovations, policy enablers and factors that constrain the governance of FLOW in the Celtic Sea. Stakeholders were invited to indicate their level of agreement with each statement. Data generated from the 14 survey responses were integrated into the case study analysis.

**Table 2:** Summary of interview participants

Stakeholder group	Code	No. interviewed
Policymakers & public bodies	PPB	4
Independent businesses	IB	4
Wind industry	WI	8
Marine users (fishing industry & eNGOs)	MU	5
Research & academia	RA	1
Local authority	LA	1
Port owners & operators	PO	1
TOTAL		**24**

### Findings

The case study findings reveal a range of maladapted institutional attributes that contribute to fragmented marine governance arrangements, rendering them ill-suited to deliver an integrated, coordinated, and targeted policy approach in support of a new FLOW industry in the UK’s Celtic Sea. Three illustrative examples of governance challenges are outlined below: ownership of governance challenges, fragmented and exclusionary knowledge regimes, and misalignments in the pace of marine policy development. These challenges are characterised by multiple institutional barriers, including policy layering, path dependency, bounded rationality, institutional inertia, unequal power relations, and inadequate stakeholder participation. These barriers were traced to ten of the 11 institutional attributes set out in the Oberlack (2017) and Nielsen et al. (2023) typology (Table 1). The paper focuses on these challenges because they consistently emerged as the most influential constraints to effective marine governance across the empirical material, shaping how authority is exercised, how knowledge is produced and mobilised, and how policy processes unfold. Together, they illuminate the interplay of agency and structure in governance processes, as each challenge reflects a distinct configuration of maladapted institutional attributes from the three key dimensions (agency, social interactions, and inherent attributes) in the analytical framework (Table 1).

#### Ownership of the ‘floating wind problem’

Institutional attributes related to actor roles and responsibilities, actor control, conflict mechanisms, social connectivity, social learning/development and use of knowledge, accountability mechanisms, and adaptiveness of institutions were found to be maladapted to the context of FLOW governance. The cross-cutting nature of FLOW requires extensive collaboration and coordination amongst actors located within and across multiple governance scales, policy domains, and sectors, yet the institutional structures and processes for ensuring a multi-level, strategic, and coordinated approach to FLOW development are weak. Interviewees from the offshore wind industry reported a lack of cross-departmental coordination on offshore wind policy and were concerned that a fragmented approach to policy development was hampering the ability of the UK to support a domestic FLOW industry and achieve net-zero goals. One interviewee stated that the policy landscape for offshore wind had developed through *“organic evolution”* (WI4) rather than through a strategic, policy design approach. Similarly, a report by the Offshore Wind Industry Council (OWIC 2024) concluded that weak coordination amongst governance actors had contributed to misalignment between different components of the offshore wind policy and legislative framework, including seabed leasing, marine spatial planning, financing mechanisms, and grid connection processes. Coordination challenges were acknowledged during an interview with a policymaker: *“Whitehall is not one big building with all the different departments*,* you know*,* we essentially operate as different entities”* (PPB1), suggesting that sectoral approaches to marine policymaking are embedded and siloed within existing institutional structures. As a result, opportunities to engage in cross-departmental coordination and learning have been missed, while attempts to address policy challenges have occurred ‘ad-hoc’ due to a lack of overall ownership of FLOW governance challenges. Hence, offshore wind policy has developed through an organic process of policy layering. Barriers arising from coordination issues are linked to institutional attributes that define procedures for connecting actors within and across tiers of governance (Social Connectivity), as well as attributes that shape how information, knowledge, values, and preferences are constructed, communicated, and accepted amongst governance actors (Social Learning/Development and Use of Knowledge). In this case, inadequate rules and procedures for facilitating meaningful coordination and collaboration on offshore wind policy across central government, devolved administrations, and public bodies have enabled siloed policymaking to persist, reinforcing governance fragmentation and shifting pressure downwards to local planning authorities who are tasked with interpreting and implementing incoherent ORE policies and strategies. Weak actor connectivity and coordination have also contributed to long-term consenting delays, which generate uncertainty and increased risk for offshore wind developers and their investors (OWIC 2024), and exacerbate tensions between competing interests (e.g. offshore wind, shipping, fishing, and nature conservation).

Institutional coordination issues were also identified in relation to marine spatial planning. At the time of the study, there were three separate marine spatial analyses underway, each being led by a different governance actor. A cross-departmental Marine Spatial Prioritisation Programme (MSPri) was being led by the Department for Environment, Food, and Rural Affairs (DEFRA); the National Energy System Operator (NESO) was developing a Strategic Spatial Energy Plan (SSEP) for the energy system (land and sea); and The Crown Estate, through its ‘Whole of Seabed Programme’, was undertaking an extensive spatial analysis to support future decision-making on seabed use. Each of these spatial analyses was taking place alongside statutory marine plan-making processes led by the Marine Management Organisation (MMO). The existence of independent parallel spatial analyses demonstrates that a diverse range of state and non-state actors are involved in marine governance, yet there is no clear delineation of roles and responsibilities, and no single body is responsible for strategic spatial planning in the maritime area. This example illustrates the fragmented nature of marine governance arrangements, which has led to siloed thinking (APPG 2021) and a lack of clarity on who has institutional responsibility for marine policy (EAC 2025). In this example, fragmentation can be traced to the maladaptation of institutional attributes that regulate the responsibilities of governance actors and the actions assigned to them (Roles and Responsibilities). The rules in use are inadequate for defining clear mandates for marine spatial analyses or making provision for institutional authority on marine policy. According to one interviewee, unclear power structures and overlapping mandates had contributed to policy incoherence:

*You have to develop relationships and talk to each of these individual departments*,* who are all doing their own little bit*,* but nobody’s got full oversight of the development*,* the ideal spatial plan as a whole*,* and consequently*,* it’s all little piecemeal bits and pieces*,* and actually some bits counteract what the other ones are trying to do* (MU3).

The absence of institutional leadership on marine policy has allowed sectoral tensions to emerge: *“You’ve got DESNZ* [Department for Energy Security and Net Zero] *making decisions that have consequences for another department’s objectives”* (MU5), and a perception amongst some marine stakeholders that sectoral approaches persist because the policy objectives of DESNZ are prioritised over nature recovery and sustainable fisheries. The dominance of a particular policy domain reflects power asymmetries between government departments and can be traced to institutional attributes that regulate the control that an actor has over governance outcomes (Control). These early signs of sectoral tensions reveal linkages between institutional attributes that shape interactions amongst actors (Social Connectivity), regulate the influence of individual actors (Control), and determine how conflicting interests among actors are resolved, transformed, or prevented (Conflict Mechanisms). In the absence of well-designed institutional attributes that define the responsibilities for institutional leadership on marine policy and allocate appropriate powers, the combination of these maladapted attributes increases the likelihood that sectoral tensions will persist.

The Crown Estate is the owner and manager of the seabed in England and Wales, and its role in marine governance was questioned during the interviews. One interview participant from the environmental NGO sector suggested that marine governance had been *“outsourced to The Crown Estate”* (MU4), which decreased levels of scrutiny and accountability due to their status as an independent profit-making entity (The Crown Estate 2025), while another participant stated that they had very little negotiating power with The Crown Estate because *“they own the sea*,* we do what they want”* (WI5). Accountability issues can be traced to the institutional rules for monitoring, evaluating, rewarding, and enforcing actor responsibilities (Accountability Mechanisms). When accountability rules are ineffective, there is a risk that governance arrangements will change in a manner that aligns with the interests of powerful actors. These kinds of concerns have been raised by the fishing industry:

*Our new government has an ambitious agenda for economic growth and social renewal. We should be talking to them about the fishing industry’s potential to contribute to this vision and to help spread its benefits to coastal communities. Instead*,* we are talking to the King’s land agents.*

(National Federation of Fishermen’s Organisations (NFFO) 2024, online)

The dominance of The Crown Estate is also reflected in several policy documents, including the SSEP Methodology (NESO 2025), which makes repeated reference to the importance of strategic coherence and alignment with The Crown Estate’s offshore leasing activities and Marine Delivery Routemap, yet it includes only a single reference to DEFRA’s MSPri Programme. Similarly, The Crown Estate’s Marine Delivery Routemap (The Crown Estate 2024) intends to *“complement and inform”* (p.18) related marine spatial analyses. These discursive representations reveal the significant influence of sectoral interests in renewable energy on marine spatial analyses and create ambiguity over which specific actors can be held accountable for spatial planning decisions. This is a further example of linkages between institutional attributes. Here, the institutional attributes of Control and Accountability Mechanisms interact to raise questions of legitimacy and trust in relation to the process and outcomes of marine spatial planning. There remains limited detail on the extent to which the actors involved in spatial planning are connected and aligned on their policy objectives, and there is evidence of institutional inertia on marine spatial prioritisation (EAC 2025). Institutional inertia can be traced to the attribute of Adaptiveness of Institutions, whereby changes to the rules-in-use on marine planning are constrained by the transaction costs associated with the need to generate new knowledge, coordinate a wider range of governance actors, and engage in stakeholder negotiations to support prioritisation decisions. This serves to reproduce existing power relations, whereby powerful actors (i.e. The Crown Estate) can drive decision-making on the allocation of marine space.

#### Fragmented and exclusionary knowledge regimes

A range of maladapted institutional attributes, including actor eligibility, actor roles and responsibilities, actor control, social learning/development and use of knowledge, and institutional incentives, contribute to the establishment of siloed knowledge production and reduced democratic participation in FLOW governance. Multiple actors are collecting marine data, and a range of digital tools and data portals are used to support FLOW planning and consenting applications and decisions. However, a lack of guidance on protocols for data standardisation has resulted in data being gathered in a *“very chaotic way”* (WI4). A similar issue was raised in a report by the Offshore Wind Champion (Pick 2023), who stated that *“a lack of alignment on baselines leads to misalignments as to impacts and compensation measures and contributes to what can become a quite adversarial (as opposed to procedural) process”.* A fragmented approach to data collection has contributed to concerns over transparency, particularly in relation to which datasets are considered legitimate ‘evidence’ and therefore accepted for use in marine planning, licensing, and permitting decisions. During the interviews, several wind industry actors expressed frustration over the lack of clarity on what constituted ‘evidence’ and pointed to missed opportunities to learn from the long history of fixed-bottom wind consenting in the UK:

*There seems to be a very*,* very high bar set to what can be regarded as a dataset or a study that can be relied upon. As a result*,* there’s quite a lot of good data and work that’s done*,* but if there’s a couple of concerns about elements of the methodology*,* it’s almost just ignored*,* rather than trying to build up a kind of macro picture* (WI8).

A fragmented ownership of process-specific knowledge (e.g. licensing processes, planning protocols, management procedures) also exists amongst government departments and public bodies. Consequently, each actor is confined to a partial view of the processes that govern the development of FLOW, and no single actor has a complete understanding of the overall process. This fragmentation limits opportunities for information sharing and cross-departmental learning on FLOW governance challenges, such as examining/determining cumulative environmental impacts and supporting co-existence between marine uses. Issues related to data fragmentation and ownership of knowledge can be traced to the institutional attributes of Roles and Responsibilities, Social Connectivity, Social Learning/Development and Use of Knowledge and Institutionalised Incentives. When these attributes function as institutional barriers, they reinforce power asymmetries and contribute to path-dependent decision-making:

*I don’t think there’s anyone really driving forward how we take all the learnings from the sector over the last two decades and apply them to making* [environmental] *assessments of the future more proportionate and focused and streamlined* (WI3).

Certain types of knowledge were marginalised during the early stages of decision-making on FLOW development in the Celtic Sea. As a result, knowledge development, particularly in relation to co-existence between uses, is occurring slowly. Interviews with fisheries sector representatives revealed a lack of engagement with the sector, leading to missed opportunities for state actors and regulators to develop a deeper understanding of fisheries data that are used for decision-making. Instead, decision-makers preferred to rely on technical data from vessel monitoring systems, GIS maps, and aggregated datasets published in scientific peer-reviewed publications. Reliance on technical datasets and scientific literature is legitimised as ‘evidence-based decision-making’, while the extensive place-based knowledge and expertise held by the fishing industry that has been built up through decades of experience at sea is treated as anecdotal. Consequently, fishing data that were used to inform the design of the Celtic Sea Leasing Round were contested by the fishing industry, which argued that the technical data integrated into marine spatial analyses were incomplete and unverified, thereby misrepresenting the impacts of FLOW on other uses of marine space:

*A couple of them* [project developers] *came to discuss with us and they put data sets up of what they thought was happening in those areas. They obviously obtained the data from somewhere*,* but I questioned the data source and our Members completely pulled it apart. It was very inaccurate* (MU1).

A report commissioned by the National Federation of Fishermen’s Organisations and the Scottish Fishermen’s Federation (ABPmer 2022) highlighted the extent of ‘spatial squeeze’ likely to be experienced by the fishing industry as a direct result of the expansion of offshore wind. However, the fishing industry is a non-statutory consultee on marine planning applications, despite being significantly impacted by changing marine policy objectives. Their non-statutory consultee status is interpreted by the fishing industry as a further indication that their knowledge is not viewed as a legitimate source of evidence for decision-making, and that powerful actors can decide whose knowledge counts as evidence and at what stage experiential knowledge should be considered in decision-making processes.

Signs of epistemic tensions were also revealed by two interview participants from the environmental NGO sector who reflected on the quality of stakeholder engagement during marine planning and marine spatial prioritisation processes, stating that *“our expertise wasn’t being listened to”* (MU3) and that stakeholders were *“brought into it at a later stage”* (MU5). These experiences contributed to a sense that less powerful actors are invited into consultation spaces *“once lines are already drawn on the map”* (MU1) and that offshore wind has been given priority over other marine policy objectives, raising questions about the transparency of marine spatial prioritisation decisions and the tokenistic nature of stakeholder participation in FLOW governance. Again, there is evidence of linkages between institutional attributes, in this case, the attributes that determine who is eligible to participate in governance (Actor Eligibility), who has control over these decisions (Control), and how different kinds of knowledge are valued, considered, and debated in governance arenas (Social Learning/Development and Use of Knowledge).

#### Misalignments in the pace of marine policy development

The pace of marine policy development is characterised by maladapted institutional attributes relating to social connectivity, actor control, scale of institutions, and adaptiveness of institutions. Since the UK General Election in July 2024, there has been a suite of policy developments that aim to streamline and speed up planning and consenting processes for offshore wind. Notable developments include planning reforms for Nationally Significant Infrastructure Projects (NSIPs), updates to the National Policy Statements (NPS) for Energy and Renewable Energy Infrastructure, and the establishment of specific measures to deliver strategic compensation. However, the broader policy architecture for offshore wind is fragmented, with the different components of that architecture developing at different speeds. For example, in contrast to offshore wind policies, DEFRA’s MSPri Programme, which is crucial for managing current and future sectoral conflicts, appears to have made limited progress. There is an absence of clear objectives or timelines for outputs (EAC 2025), despite widespread recognition of the need for a more strategic approach to the use of marine space (ABPmer 2022; Wildlife and Countryside Link 2023; OEP 2025). This is indicative of a temporal scale mismatch, whereby FLOW projects are already progressing, yet the policy architecture to support sustainable marine development is still being designed. This mismatch is rooted in the institutional attributes that determine the appropriate spatial and temporal scales for addressing a particular governance challenge (Scale of Institutions). One interview participant suggested that the apparent institutional inertia on marine spatial prioritisation was due to an unwillingness to make politically sensitive decisions because of the risk of legal challenges: *“They essentially just don’t make a decision and let inertia take its course”* (MU4). As a result, marine planners are ‘locked-in’ to an outdated approach that focuses on balancing the different uses of marine space instead of prioritising them in line with broader policy goals. This is a further example of path-dependency, stemming from the attributes of institutions that determine the ability to adapt and implement new governance approaches (Adaptiveness of Institutions).

Policy acceleration around offshore wind planning and consenting has also created a temporal mismatch with statutory marine planning processes. English Marine Plans are subject to a three-year review cycle followed by Parliamentary approval for a plan update. A subsequent plan update can take two to three years. The current South-West Marine Plan (MMO 2021) lacks a specific policy provision for FLOW, yet the first three-year review of the plan (DEFRA 2025) concludes that the plan does not need to be revised and will be retained in its current state. Therefore, the prospect of a South-West Marine Plan that adequately plans for FLOW is several years away, with the FLOW projects in Leasing Round 5 likely to be well through the planning and consenting process and possibly into the early phases of construction before the South-West Marine Plan is updated with a specific (and retrospective) policy and spatial plan for FLOW. Reflecting on this temporal mismatch, two interview participants described the South-West Marine Plan as *“not fit for purpose”* (WI4 and MU3). As noted in a report by the RSPB (2022, pg 2), *“*[Marine] *planning systems have failed to keep pace with the evolution and scale of offshore wind”.*

The temporal mismatch in policy development is exacerbated by the timeframes at which evidence for decision-making is produced, evaluated, and accepted. Statutory Nature Conservation Bodies (SNCBs) are required to assess large volumes of evidence in the consenting process and rely heavily on peer-reviewed publications to prepare consenting guidance. The wind industry also needs access to research outputs to support its Environmental Impact Assessments. However, the time taken to publish scientific data is mismatched with the timelines of project deployment. A meeting of the Offshore Wind Evidence and Change Programme (OWECP) Steering Group in September 2023 noted that *“industry deployment of offshore wind*,* or requirement for evidence*,* moves faster than the science programme*,* reducing the window for impact”* (OWECP 2023). According to the offshore wind industry, the precautionary approach adopted by SNCBs also contributes to temporal mismatches and leads to path-dependent decision-making and missed opportunities to shorten consenting timeframes:

*There’ll be studies that were completed*,* you know*,* four or five years ago that show a demonstrable reduction compared to our assumptions around for example*,* bird mortality and they are still not regarded by the stakeholders and regulators as being the state of the art. They’re still relying on even more conservative*,* less well-informed assumptions from*,* you know*,* eight*,* 10*,* 12 years ago* (WI8).

Misalignment in the pace of policy development reveals a lack of coordination between government departments that have a role in FLOW development. It also indicates that energy policymakers (i.e. DESNZ) are powerful actors in the governance arrangement and are therefore able to advance their policy objectives more easily than policymakers focused on environmental protection and nature recovery. For example, the development of a Marine Net Gain (MNG) policy by DEFRA that will advance nature recovery objectives is progressing slowly:

*Since the publication of the government’s response on the 9 December 2023*,* there has been no official communication about the progress made on developing the MNG proposal. More than a year later*,* we still do not know what the MNG regime will look like* (Szij 2025; online).

The UK Government has also failed to achieve Good Environmental Status (GES) by 2020, a requirement under regulation 4(1) of the Marine Strategy Regulations 2010, which has resulted in an investigation into DEFRA by the Office for Environmental Protection (OEP 2025). As these examples illustrate, institutional attributes that determine governance scales (Scale of Institutions) are linked with attributes that connect actors within networks (Social Connectivity) and regulate the allocation and distribution of power (Control). The slower pace of environmental policy development risks policy incoherence and hinders understanding of the potential for co-existence between marine uses. It also creates an impression amongst less powerful actors that they are being excluded from marine spatial prioritisation decisions. Therefore, a lagging environmental policy framework serves to postpone the management of spatial conflicts rather than ensure they are addressed and/or prevented early in the development of FLOW projects.

## Discussion

The increasing demand for marine resources and the industrialisation of ocean space through the green transition (Bilas et al. 2022) and ‘blue growth’ paradigms (Wright 2015) requires new and innovative governance arrangements that can deliver on multiple policy objectives, while enhancing ocean sustainability and delivering a just and sustainable low-carbon future (Brodie Rudolph et al. 2020; Lange and Cummins 2021; van Leeuwen et al. 2025). The UK has made a strong commitment to the low-carbon transition, underpinned by legally binding targets to achieve net-zero emissions by 2050, as well as ambitious targets for 43–50 GW of offshore wind by 2030, including 5GW of FLOW. However, ORE developments present specific governance challenges, including the need to coordinate actors within and across governance scales, manage spatial conflicts between marine uses, and ensure meaningful and inclusive stakeholder participation in governance processes. This study examined the ability of existing marine governance arrangements to change and innovate in response to these challenges. Drawing on theories and frameworks of institutional change and adaptation (e.g. Ostrom 2005; Oberlack 2017; Nielsen et al. in prep), the study identified a range of governance challenges, including institutional ambiguity over FLOW development challenges, poorly integrated and exclusionary knowledge systems, and misalignments in the pace of marine policy developments across sectors, that constrain opportunities for governance change and innovation in support of a new FLOW industry in the UK’s Celtic Sea. Using a diagnostic approach, these challenges were traced to multiple and interacting institutional attributes that were maladapted to the governance context. Although the UK is widely regarded as a global leader in FLOW development and is making significant advances in terms of commercialising FLOW technologies, the maladaptation of institutional attributes hinders the ability of governance actors to overcome these challenges and deliver on offshore wind targets and broader net-zero ambitions. Thus, the study highlights the role of institutional attributes in shaping the capacity of governance arrangements to meet complex societal challenges.

Fragmentation is a common feature of marine governance arrangements, indicating widespread and deep-rooted institutional design issues. Designing effective institutional attributes for marine governance is challenging because of the diverse range of activities that take place at sea, with no single authority having responsibility for the management and governance of ocean space (van Tatenhove 2013). The fragmented and complex nature of marine governance in England is well documented (i.e., Boyes and Elliot 2015, 2016), and this study demonstrates that more than a decade later, fragmentation persists. Furthermore, governance of the UK’s Celtic Sea spans the jurisdictions of England and Wales, involving a diverse set of decision-makers located across multiple scales of governance. While there is broad strategic alignment in the approach to offshore wind across England and Wales, devolution has resulted in diverging policy frameworks for ORE planning and licensing, as well as uneven institutional capacities and dependencies. For example, the Welsh Government’s offshore wind ambitions remain closely tied to decisions made at UK Government level, including the establishment of national targets, policy and regulatory frameworks that inform seabed leasing design, grid planning and development, financing mechanisms (e.g. Contracts for Difference), and spending decisions that affect Welsh government budgets (WAC 2023). These dependencies shape how governance operates across scales and can slow coordination, create misalignment between leasing, planning, and funding cycles, and give rise to tensions between governance actors. Thus, poorly designed institutional attributes create challenging conditions for collective decision-making and amplify unequal power relations in multi-level settings (van Tatenhove 2016). Realising UK-wide net-zero ambitions will therefore require deliberate efforts to build institutional linkages and foster joint strategic planning across governance scales in pursuit of a common vision for the emerging FLOW industry. Efforts are being made in this direction, exemplified by the collaboration between the Welsh Government and the MMO to develop a guide for cross-border marine planning in the Severn Estuary, and the establishment of the Welsh Government’s Offshore Wind Task and Finish Group, which explicitly recognises that delivery of FLOW in the Celtic Sea depends on cross-scale collaboration. The formation of ‘Mission Boards’ by the Labour Government following their election in 2024 also acknowledges the need for new governance mechanisms to overcome siloed thinking (Diamond et al. 2025). This governance development may offer a partial remedy to fragmentation by improving coordination between central government departments and public bodies. However, there is also a risk that the Mission Boards contribute to government ‘thickening’ (van der Heijden 2011). This kind of institutional layering at the highest scale of governance could contribute to a further overlap of responsibilities and reduce transparency over FLOW development decisions.

As well as fragmentation, maladapted institutional attributes precipitated a range of other governance challenges in the Celtic Sea, related to institutional authority, development and use of knowledge, and the pace of policy development. These challenges revealed evidence of policy layering, path dependency, bounded rationality, institutional inertia, unequal power relations, and inadequate stakeholder participation, which can be recast as *symptoms* of maladapted institutional attributes, as they reflect the outcomes of structural or procedural constraints that are embedded within existing interactions, practices, and power relations. Examples include fragmented and overlapping roles and responsibilities; the existence of boundary rules that restrict access to decision-making arenas; power asymmetries amongst government departments; lack of coordination amongst governance actors; inadequate conflict resolution mechanisms; limited knowledge coordination and learning; inadequate accountability mechanisms; temporal scale mismatches; and low institutional adaptiveness, with many of these operating synergistically (Eisenack et al. 2014). In this study, weak actor connectivity, control of governance agendas by powerful actors, and inadequate structures and processes to support social learning were the most prevalent barriers. These findings align with a scoping review of institutional barriers in a European maritime context (Nielsen et al. 2023), in which institutional attributes shaping actor control and the development and use of knowledge were frequently reported barriers. Similarly, in a meta-analysis of climate adaptation across Europe (Oberlack 2017), weak social connectivity amongst governance actors was a particularly frequent barrier. The cross-cutting nature of climate adaptation generates an inter-dependency between governance actors, and hence, high levels of actor connectivity and coordination are required. Similar interdependencies are evident in FLOW development, which cuts across multiple policy domains. Therefore, without high levels of strategic coordination and cooperation across scales, as well as a shared vision across policy domains and sectors, challenges related to the governance of new industrial developments at sea, including managing sectoral conflicts, are likely to persist (Kusters et al. 2025).

An institutional lens provided a useful approach for understanding the origins of institutional barriers and their relationship with particular attributes of institutions. Through the adoption of a novel diagnostic approach, the institutional barriers identified were traced to ten of the 11 institutional attributes set out in the Oberlack (2017) and Nielsen et al. (2023) typology. A diagnostic approach enabled a more systematic examination of institutional barriers that focused on the linkages and synergies between them that shape their emergence and persistence. Institutional attributes give rise to institutional barriers when they become misaligned with governance needs. This misalignment has likely emerged as a result of the rapid changes taking place at sea over the past two decades, in which the type and intensity of maritime uses have soared (Schupp et al. 2019), as well as global climate impacts, which present an evolving challenge for policymakers (Santos et al. 2016). Current marine governance arrangements are designed for a past policy context focused on sectoral management (Stephenson et al. 2019) and have failed to transform and innovate in a manner that can deliver the integrated, holistic approaches that are needed to address contemporary, cross-sectoral challenges, including climate change, biodiversity loss, and the low-carbon transition. Together, the institutional barriers identified in this study reveal how established rules, norms, and practices are resistant to reform even as new marine policy objectives emerge, pointing to a broader problem of institutional rigidity in the face of complex, cross-sectoral governance challenges. The UK Government’s approach to evidence-based decision-making is indicative of this institutional rigidity. Despite new commitments to a comprehensive and informed approach to marine management and decision-making, marine decisions continue to rely heavily on specific evidence types, such as data and statistics, maps, and modelling outputs, while excluding evidence from stakeholders with local place-based knowledge and experiences (Fairbrass et al. 2025). Thus, overcoming institutional barriers requires more than technical fixes; it involves reconfiguring governance arrangements to support reflexivity, adaptability, and collaborative learning (van Leeuwen et al. 2025).

Given the central role of ORE development in advancing the low-carbon transition at both national and international scales, overcoming institutional barriers must be considered a key imperative for governance actors. Here, enduring institutional issues such as policy layering, path dependency, and institutional inertia are reconceptualised as symptoms of more deep-rooted institutional maladaptations rather than discrete barriers in their own right. This more nuanced understanding of institutional barriers implies that the ability of governance actors to overcome institutional barriers and transform policy and decision-making processes is dependent not only on their governance capabilities and access to social learning opportunities within their organisations, but also on the origin(s) of the barrier itself (Moser and Ekstrom 2010). Clarifying institutional mandates is an essential starting point for reducing ambiguity over actor roles and responsibilities, strengthening accountability, and curbing the dominance of powerful actors in governance arenas. Equally important is the development of institutional structures and processes that incentivise data sharing, foster cross-sector learning and collaboration, and support diverse knowledge inputs into governance processes. As noted by Vella and Baresi (2017), policy actors can overcome barriers when the design of institutional attributes allows space for improvisation and discretion while responding to different types of conflicts and challenges.

The transition to a low-carbon society is increasingly recognised as a deeply institutional, political, and socio-economic process (Andrews-Speed 2016). The findings from the Celtic Sea case support this proposition, revealing how institutional attributes are shaping the speed and direction of the low-carbon transition. While this paper focuses on England and Wales, comparable institutional barriers are evident across European energy transitions. In Denmark, for example, ambitions to develop large-scale artificial energy islands have been delayed by numerous barriers, including uncertainty about actor roles and responsibilities, control of decision-making by high-level political actors, and inadequate involvement of key stakeholders (Dyremose 2025). Furthermore, the Danish energy islands have the status of ‘Project of Common Interest’, a European Union initiative aimed at accelerating key cross-border energy projects. However, managing tensions between EU ambitions, national priorities, and local impacts has presented a formidable scale-related governance challenge (Dyremose et al. 2026). Beyond Europe, fragmented authority, weak coordination between jurisdictions, and political uncertainty have contributed to offshore wind project delays in the United States (Hansen et al. 2026), while China’s rapidly expanding offshore wind sector is hindered by poorly designed institutional attributes, with provisions for renewable energy scattered across various central and local level laws, and within general laws, specialised laws and administrative rules (Liu 2019). Thus, the governance challenges in the Celtic Sea reflect a broader pattern: energy transitions unfold within complex transboundary, multi-scalar institutional environments, where national ambitions depend on cooperation across multiple scales, jurisdictions, sectors, and interests.

Recent geopolitical events have caused energy security shocks, contributing to a shift in global discourses on renewable energy, framing it not only as a climate mitigation strategy but also as a means of securing affordable, resilient energy systems and creating economic value (Gibbs and Jenson 2022). However, amidst heightened geopolitical tensions, these discourses surface deeper tensions around maritime jurisdictions, environmental governance, and access to critical minerals that are essential for offshore wind development (Lee et al. 2024). Thus, the UK’s FLOW ambitions are increasingly embedded within global political–economic discourses, whereby external geopolitical crises are shifting policy focus away from long-term sustainability goals. Taken together, these evolving discourses underscore how institutional arrangements ultimately determine which visions of the energy transition secure wider acceptance and how they are translated into policy and practice.

## Conclusion

Understanding the linkages between institutional attributes and barriers is critical for designing effective solution pathways that enable more transformative and adaptive forms of marine governance. By examining how specific institutional features—such as rules, norms, power relations, and coordination mechanisms—shape the behaviour of governance actors, it becomes possible to identify the structural and procedural constraints that limit governance change and innovation. Recognising these underlying connections helps to shift the focus from treating symptoms, such as fragmentation or inertia, towards addressing the root causes of institutional maladaptation. This diagnostic perspective supports the development of targeted interventions that realign institutional attributes with sustainability objectives, enhance cross-sectoral coordination, and strengthen the adaptive capacity of governance systems. In the context of FLOW development in the Celtic Sea, this requires the development of governance solutions that embed multi-scalar collaborative approaches and incorporate diverse forms of knowledge and expertise. Ultimately, such an approach is essential for achieving dual climate and nature goals and supporting a just transition to a low-carbon society.

The use of a diagnostic approach in future research on marine governance, particularly in contexts where intensifying pressures from both traditional and emerging marine sectors heighten the risk of spatial conflicts and policy incoherence, would be useful. The Celtic Sea case demonstrates that structural and procedural constraints are often deeply entrenched and can restrict the scope for governance change and innovation, even in progressive policy contexts. An early diagnosis and understanding of the root causes of institutional maladaptation is therefore essential for identifying strategic actions to overcome them.

## Supplementary Information

Below is the link to the electronic supplementary material.

Supplementary Material 1
