# Supplementary material for: Beyond the surface: diagnosing the origins of institutional barriers in the governance of floating offshore wind
Source: Marit Stud. 2026 Jun 30;25(3):40. doi: 10.1007/s40152-026-00499-4 (PMC13320046; doi:10.1007/s40152-026-00499-4)
Supplement: Supplementary file 1 — Supplementary Material 1 [file 40152_2026_499_MOESM1_ESM.pdf]

Supplementary Information

Maritime Studies

BEYOND THE SURFACE: DIAGNOSING THE ORIGINS OF INSTITUTIONAL BARRIERS IN THE GOVERNANCE OF FLOATING OFFSHORE WIND

[1]Lindsey West, Christina Kelly, Wesley Flannery

[1]School of Natural and Built Environment, Queen's University Belfast, Belfast, BT9 5AG, UK

Email of corresponding author: l.west@qub.ac.uk

Table S1: List of documents reviewed and analysed

Document Type	Title	Year	Author	Link
Government statement	Governing the marine environment: Government response	2025	UK Government	Governing the marine environment: Government Response
Report	Marine Governance	2025	Environmental Audit Committee	https://committees.parliament.uk/publications/48204/documents/252309/default/
Technical document	Strategic Spatial Energy Plan (SSEP) Methodology	2025	National Energy System Operator	https://www.neso.energy/document/360501/download
Consultation response	Wildlife and Countryside Link Evidence to the Environmental Audit Committee on Governing the Marine Environment	2025	Wildlife & Countryside Link	https://www.wcl.org.uk/docs/EAC_inquiry_-_Governing_the_Marine_Environment_-_Wildlife_and_Countryside_Link_Response_-_FINAL.pdf
Position statement	Guiding principles for Marine Spatial Planning	2025	Wildlife & Countryside Link	Guiding_principles_for_Marine_Spatial_Planning_30.11.23.pdf
Position statement	The Crown Estate Bill: Committee Stage Briefing	2025	Wildlife & Countryside Link	Crown_Estate_Bill_Committee_Stage_Briefing.pdf

Position statement	The Crown Estate Bill – Briefing for Second Reading	2025	Wildlife & Countryside Link	https://www.wcl.org.uk/docs/assets/uploads/The_Crown_Estate_Bill_2nd_Reading_Briefing_from_Link.pdf
Meeting minutes	Welsh Marine Planning Stakeholder Reference Group meeting: 1 July 2025	2025	Welsh Marine Planning Stakeholder Reference Group	https://www.gov.wales/marine-planning-stakeholder-reference-group-meeting-1-july-2025-html
Meeting minutes	Welsh Marine Planning Stakeholder Reference Group meeting: 19 March 2025	2025	Welsh Marine Planning Stakeholder Reference Group	Marine Planning Stakeholder Reference Group meeting: 19 March 2025 [HTML] | GOV.WALES
Report	Three-year report on the South West Inshore and South West Offshore Marine Plan	2025	Department for Environment, Food and Rural Affairs	Three-year report on the South West Inshore and South West Offshore Marine Plan
News release	OEP launches investigation into a suspected failure by Defra to take the necessary measures to achieve Good Environmental Status (GES) of marine waters	2025	Office for Environmental Protection	https://www.theoep.org.uk/news/oep-launches-investigation-suspected-failure-defra-take-necessary-measures-achieve-good
Online blog	Marine Net Gain Proposal (2022): An Overview	2025	Csongor Szij (Richard Buxton Solicitors)	Marine Net Gain Proposal (2022): An Overview - Richard Buxton Solicitors
Policy brief	Planning for energy: Challenges and solutions for local authority skills and resourcing	2025	ReGen	https://cdn.prod.website-files.com/6798bfee7da7f37163ec22fb/687f731bc7bf0d769d4131c6_20250722%20Local%20planning%20authority%20skills%20and%20resourcing%20policy%20brief%20-%20Regen.pdf
Consultation response	Planning for New Energy Infrastructure	2025	ReGen	https://cdn.prod.website-files.com/6798bfee7da7f37163ec22fb/6842fb5072261d8bbe15bbbc_2025-06-06%20-%20Response%20to%20NPS%20revisions%20-%20Regen.pdf
Policy document	National Policy Statement for Energy - Update 2025	2025	Department for Energy Security and Net Zero	Appraisal of Sustainability – Non-Technical Summary
Report	Standardisation of Post-Consent Environmental Monitoring for Wind Farms in English Waters	2025	Marine Management Organisation	https://assets.publishing.service.gov.uk/media/68776d6e0263c35f52e4dc25/Offshore_Wind_Monitoring_Standardisation_Report.pdf

Statutory guidance	Standardisation of Post-Consent Environmental Monitoring for Wind Farms in English Waters	2025	Marine Management Organisation	Offshore_Wind_Monitoring_Standardisation_Guidance.pdf
Position statement	Reflections from UK Marine Industries on the Planning and Infrastructure Bill	2025	Seabed User & Developers Group	https://www.sudg.org.uk/post/reflections-from-uk-marine-industries-on-the-planning-and-infrastructure-bill
Meeting presentation	Offshore Wind Evidence & Change Programme - Programme Steering Group Meeting Feb 2024	2024	Offshore Wind Evidence & Change Programme Steering Group	https://assets.ctfassets.net/nv65su7t80y5/17fv11VYWAJyHXiPCypbma/6f260cdd6e57ecb1e74f0366b6be57ed/OWEC_PSG_Feb_2024_Presentation_Slides.pdf
Meeting presentation	Offshore Wind Evidence & Change Programme - Programme Steering Group Meeting May 2024	2024	Offshore Wind Evidence & Change Programme Steering Group	Offshore Wind Evidence + Change Programme
Meeting presentation	Offshore Wind Evidence & Change Programme - Programme Steering Group Meeting Sept 2024	2024	Offshore Wind Evidence & Change Programme Steering Group	1727340064-owec-psg-september-2024-slide-deck.pdf
Report	The Missing Middle	2024	Celtic Sea Power	https://celticseapower.co.uk/wp-content/uploads/2023/12/CSP-Missing-Middle-PR5.pdf
Position statement	RWE response to confirmation of Allocation Round 6 (AR6) budgets and parameters	2024	RWE	https://uk.rwe.com/press-and-news/uk-statements-and-opinion/2024-03-07-rwe-response-to-confirmation-of-allocation-round-6-budgets-and-parameters/
House of Commons Parliamentary transcript	Floating offshore wind	2024	Hansard	https://hansard.parliament.uk/commons%E2%80%8F/2024-12-12/debates/B84D9DEF-A9C2-487A-B61B-8CF561C152BB/FloatingOffshoreWindCelticSea
Report	Policy and Legislative Barriers to Offshore Wind Consenting	2024	Offshore Wind Industry Council	https://www.owic.org.uk/media/itrporhd/5314-culpepper-owic-policy-and-legislation-report-v9.pdf
Report	Use of evidence and data in decision-making<br>in offshore wind farm consenting	2024	Offshore Wind Industry Council	https://www.owic.org.uk/media/4zldf4zq/use-of-evidence-and-data-in-decision-making-in-offshore-wind-farm-consenting_final.pdf
Report	Marine Delivery Routemap	2024	The Crown Estate	https://www.datocms-assets.com/136653/1742378449-marine-delivery-routemap-2024.pdf

Report	Marine Delivery Routemap: Future of Offshore Wind	2024	The Crown Estate	https://www.datocms-assets.com/136653/1742378325-future-offshore-wind-2024.pdf
Report	Whole of Seabed Programme Methodology	2024	The Crown Estate	https://www.datocms-assets.com/136653/1720789831-whole-of-seabed-summary-methodology-report-2024.pdf
Meeting minutes	Welsh Marine Planning Stakeholder Reference Group meeting: 02 October 2024	2024	Welsh Marine Planning Stakeholder Reference Group	https://www.gov.wales/marine-planning-stakeholder-reference-group-meeting-2-october-2024-html
Online blog	The Crown Estate's Marine Routemap: The rush to develop offshore power.	2024	National Federation of Fishermen's Organisations	https://www.nffo.org.uk/the-crown-estates-marine-routemap-the-rush-to-develop-offshore-power/
Report	Marine Planning for Nature in Wales	2024	Marine Conservation Society	Marine_Planning_for_Nature_in_Wales_-_2024.pdf
Report	Evidence Review: Drivers and Pressures Affecting the UK Marine Environment	2024	Office for Environmental Protection	OEP - Evidence Review Report - Drivers and Pressures Affecting the UK Marine Environment V2.pdf
Policy document	National Planning Policy Framework	2024	Ministry of Housing, Communities and Local Government	https://assets.publishing.service.gov.uk/media/67aafe8f3b41f783cca46251/NPPF_December_2024.pdf
Report	Delivering the shared offshore network	2024	Offshore Wind Industry Council	offshore-grid-coordination-report.pdf
Policy document	Clean Power 2030 Action Plan	2024	Department for Energy Security and Net Zero	Clean Power 2030: Action Plan: A new era of clean electricity
Meeting presentation	Offshore Wind Evidence & Change Programme - Programme Steering Group Meeting Feb 2023	2023	Offshore Wind Evidence & Change Programme Steering Group	20230208-owec-psg-feb-2023-slides-final.pdf
Meeting presentation	Offshore Wind Evidence & Change Programme - Programme Steering Group Meeting Sept 2023	2023	Offshore Wind Evidence & Change Programme Steering Group	https://assets.ctfassets.net/nv65su7t80y5/5eBA5OQZakb5zH4PuTRmBS/a83aae97399ab1785b1a8d04e5cd105b/psg-meeting-website-powerpoint-sept-2023.pdf
Meeting minutes	Port Finance Roundtable	2023	Celtic Sea Power	Port Finance Roundtable 28th September 2023 - Notes

Report	Independent report of the Offshore Wind Champion: Seizing our Opportunities	2023	Tim Pick	https://assets.publishing.service.gov.uk/media/65a662c1867cd800135ae90b/offshore-wind-champion-independent-report.pdf
House of Commons Parliamentary transcript	Floating offshore wind	2023	Hansard	https://hansard.parliament.uk/commons/2023-11-16/debates/18C8975F-ACEE-410F-95DC-4EF4B164D6B6/FloatingOffshoreWind
Information document	Information Memorandum: Celtic Sea Floating Offshore Wind Leasing Round 5	2023	The Crown Estate	Information_Memorandum.pdf
Policy document	National Policy Statement for Energy (EN-1)	2023	Department for Energy Security and Net Zero	https://assets.publishing.service.gov.uk/media/65bbfbdc709fe1000f637052/overarching-nps-for-energy-en1.pdf
Policy document	National Policy Statement for Renewable Energy Infrastructure (EN-3)	2023	Department for Energy Security and Net Zero	https://assets.publishing.service.gov.uk/media/65a7889996a5ec000d731aba/nps-renewable-energy-infrastructure-en3.pdf
Position statement	Delivering for 2035: Upgrading the grid for a secure, clean and affordable energy future	2023	National Grid	https://www.nationalgrid.com/document/149501/download
Report	Floating Offshore Wind in Wales. Second Report of Session 2022–23.	2023	House of Commons Welsh Affairs Committee	https://committees.parliament.uk/publications/34262/documents/188581/default/
Position statement	Considerations for Marine Spatial Prioritisation<br>Decision Making in the UK	2023	Seabed User & Developers Group	https://www.sudg.org.uk/blog/page/3
Report	Powering Healthy Seas	2022	RSPB	powering-healthy-seas-report_rspb_august-2022.pdf
Report	Spatial Squeeze in Fisheries	2022	APBmer	https://www.nffo.org.uk/the-frightening-outlook-of-fisheries-displacement-spatial-squeeze-report-published/
House of Commons Parliamentary transcript	Floating offshore wind projects	2022	Hansard	https://hansard.parliament.uk/commons/2022-10-18/debates/0C546F84-0C75-47CD-B985-63B0A6854836/FloatingOffshoreWindProjects
Policy document	British Energy Security Strategy	2022	Department for Business, Energy, & Industrial Strategy	https://assets.publishing.service.gov.uk/media/626112c0e90e07168e3fdba3/british-energy-security-strategy-web-accessible.pdf

Report	Accelerating planning to deliver a Net Zero energy system	2022	Energy UK	EnergyUKReport_Acceleratingplanningtodeliveranetzeroenergysystem_June2022.pdf
Policy brief	UK fishing and offshore wind	2021	All-Party Parliamentary Group on Fisheries	Policy Brief: UK Fishing and Offshore Wind
Report	Virtual Floating Offshore Wind Project Planning Exercise	2021	National Federation of Fishermen's Organisations	https://www.marinedataexchange.co.uk/details/TCE-3664/2021-2023-national-federation-of-fishermens-organisations-offshore-wind-evidence-and-change-programme-owec-virtual-floating-offshore-wind-fow-project
Policy document	South West Marine Plan	2021	Marine Management Organisation	https://www.gov.uk/government/publications/the-south-west-marine-plans-documents
Policy document	Net Zero Strategy	2021	Department for Business, Energy, & Industrial Strategy	https://assets.publishing.service.gov.uk/media/6194dfa4d3bf7f0555071b1b/net-zero-strategy-beis.pdf
Report	Industrial leadership – Unlocking the UK's floating wind potential	2021	Offshore Renewable Energy Catapult	https://fowcoe.co.uk/wp-content/uploads/2023/09/7527-Catapult-Report-%E2%80%93-Industrial-Leadership-FINAL.pdf

Table S2: Survey statements embedded in ArcGIS StoryMap, with 5-point Likert scale response options (Strongly Agree/Agree/Neither Agree or Disagree/Disagree/Strongly Disagree)

Statement theme	Statement
Change and Innovation: Transformative seabed leasing design	Early stakeholder engagement in Leasing Round 5 created multiple opportunities for a diverse range of sea users to engage in the seabed leasing design process. It also signposted The Crown Estate to data that may otherwise have been overlooked. Early engagement was beneficial because it helped build an understanding of the areas of interest long before the leasing stage, and allowed challenging issues to be identified, raised, and discussed at the outset, thereby reducing the potential for conflict during consultation stages.
Change and Innovation: A new policy presumption	The new policy guidance which considers offshore wind developments as Critical National Priority infrastructure has received a mixed response. The offshore wind industry welcomes the new designation because it will speed up planning and consenting decisions and help deliver on the 2030 floating wind targets. The environmental sector is concerned about the risks to vulnerable species and habitats if the urgent need for renewable energy is presumed to outweigh the environmental impacts of floating wind projects.
Enabling policy	Specific floating wind targets connect government ambitions with broader net zero commitments. Accelerated targets are being driven primarily by energy security concerns.
Enabling policy	The Contracts for Difference (CfD) scheme has been extremely successful at providing a route to market for fixed bottom wind and is a vital funding mechanism for the commercialisation of floating wind. However, the CfD mechanism should be redesigned to remove the competitive auction environment and help secure a steady growth in the floating wind project pipeline towards the 2030 targets.
Enabling actors	State actors have developed a broad suite of policies that support the development of floating wind, although policy implementation could be improved. The establishment of GB Energy suggests that the new Labour government recognises the crucial role of the State in developing a new floating wind sector, rather than being dependent on market forces to drive the sector forward.
Enabling actors	The Crown Estate is funding a range of initiatives that support the establishment of a floating wind sector and therefore plays an enabling role that goes well beyond seabed leasing.
Constraining policy	The English marine planning system is widely perceived to be inadequate for managing increasing pressures on marine space. Specifically, there is no clear mechanism within marine planning processes for prioritisation of different marine uses or for co-existence between uses, which increases the risk of conflict between nature conservation objectives, fisheries management, shipping and navigation, and offshore wind. Weaknesses in the institutional arrangements for marine planning in England increase the risk that achieving ambitious floating wind targets will result in negative environmental impacts.

Constraining policy	The complex policy and legislative landscape that underpins planning and consenting processes, including the inadequacy of the marine planning system, means that SNCBs are required to make consenting decisions without appropriate levels of policy guidance, particularly in terms of cumulative impacts of offshore wind developments. The lack of policy guidance has contributed to an increase in instances of derogations for offshore wind projects and significant delays to project consenting.
Constraining delivery environment	Specific targets and timeframes for floating wind and net zero demonstrate government ambition, but there is no credible roadmap for achieving these targets. Decision-makers are spread across multiple government departments with limited connectivity between them. Marine policies are developing in silos and there is limited knowledge sharing between institutions and organisations. The absence of a roadmap creates uncertainty for investors, reduces the likelihood that a new floating wind sector will deliver economic and social value to the UK, and risks further misalignment between climate and nature goals.
Transboundary governance	The establishment of a new floating wind sector in the Celtic Sea provides a unique opportunity for the UK, Ireland and France to develop a strategic and coordinated sea basin-level approach to floating wind development. Long-term planning for shared infrastructure would minimise environmental impacts as well as impacts on other users of the sea. However, a transboundary approach has been complicated by Brexit and is not a priority for the UK.
